# Supplementary material for: Caecilian Genomes Reveal the Molecular Basis of Adaptation and Convergent Evolution of Limblessness in Snakes and Caecilians
Source: Mol Biol Evol. 2023 May 18;40(5):msad102. doi: 10.1093/molbev/msad102 (PMC10195157; doi:10.1093/molbev/msad102)

Supplementary Figure S2: Alignment of ZRS enhancer region across a range of vertebrates illustrating the loss of an otherwise well conserved ZRS region in snakes and caecilians.

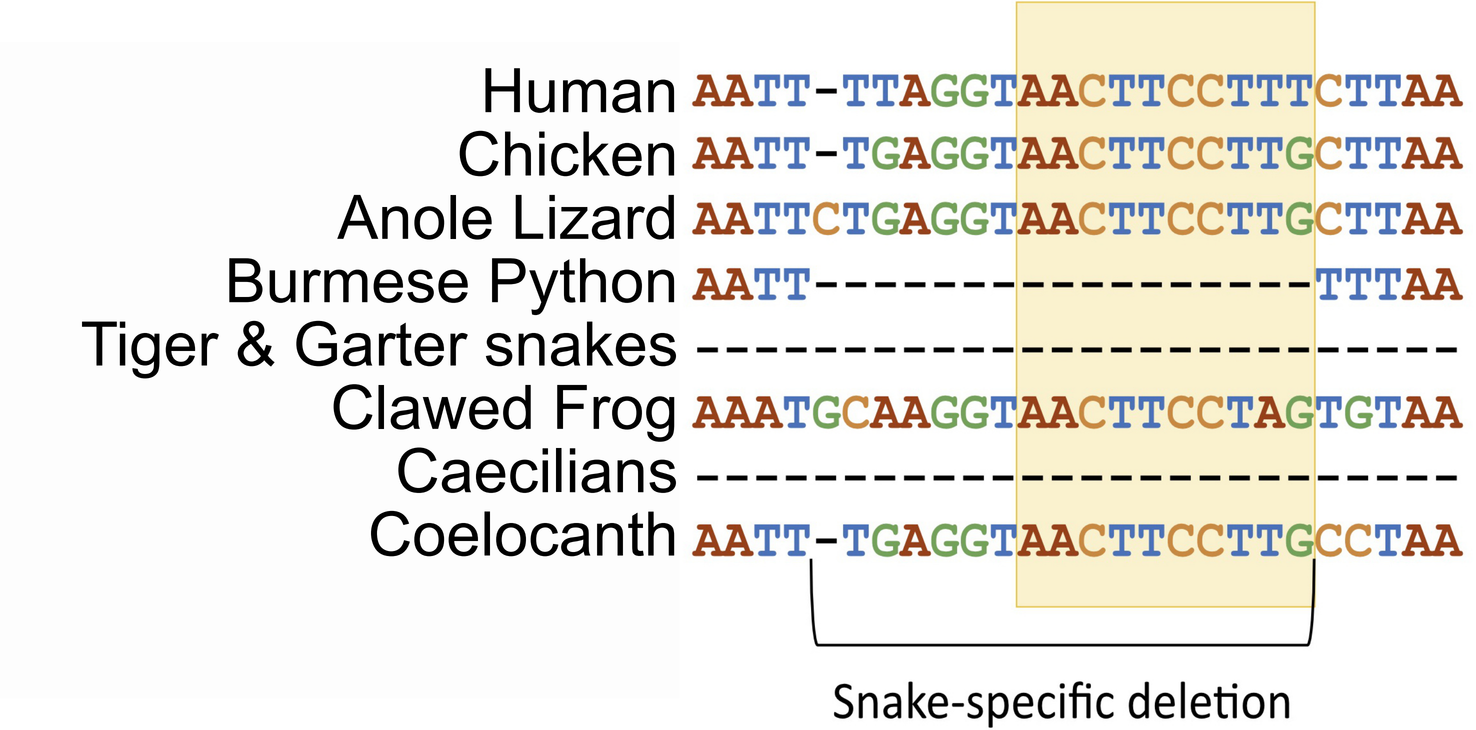

Supplement: msad102_Supplementary_Data [file msad102_supplementary_data.zip › Supplementary_Figure_S2.pdf]
