# Supplementary figures and images for: Caecilian Genomes Reveal the Molecular Basis of Adaptation and Convergent Evolution of Limblessness in Snakes and Caecilians
Source: Mol Biol Evol. 2023 May 18;40(5):msad102. doi: 10.1093/molbev/msad102 (PMC10195157; doi:10.1093/molbev/msad102)

a

### VGP assembly standard pipeline (v1.0 ~ v1.6)

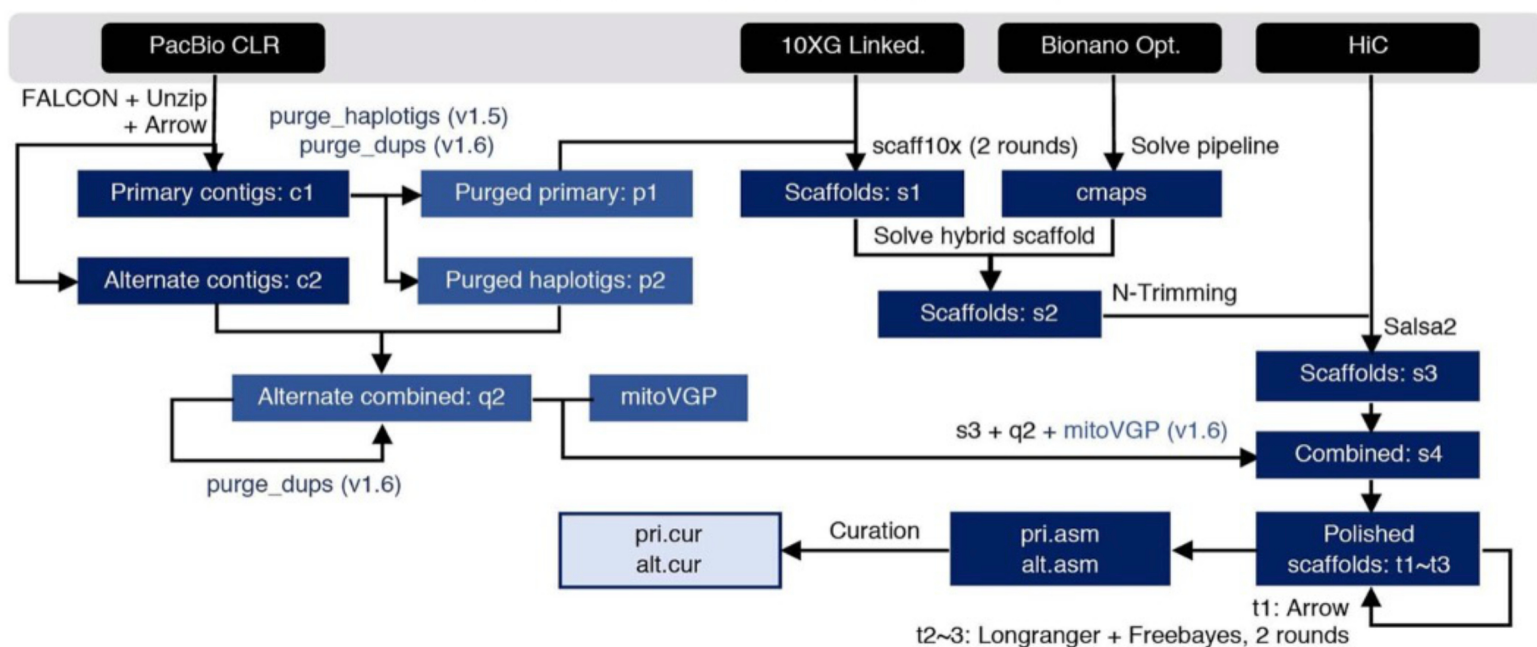

b

Taken from Rhie et al, 2021

Supplement: msad102_Supplementary_Data [file msad102_supplementary_data.zip › Supplementary_Figure_S1.pdf]
